# Divergent Retention of Sucrose Metabolism Genes after Whole Genome Triplication in the Tomato (*Solanum lycopersicum*)

**DOI:** 10.3390/plants12244145

**Published:** 2023-12-13

**Authors:** Yang Xu, Zhuping Yao, Yuan Cheng, Meiying Ruan, Qingjing Ye, Rongqing Wang, Guozhi Zhou, Jia Liu, Chaochao Liu, Hongjian Wan

**Affiliations:** 1 State Key Laboratory for Managing Biotic and Chemical Threats to the Quality and Safety of Agro-Products, Institute of Vegetables, China-Australia Research Centre for Crop Improvement, Zhejiang Academy of Agricultural Sciences, Hangzhou 310021, China; 2021720836@yangtzeu.edu.cn (Y.X.); yaozp@zaas.ac.cn (Z.Y.); chengyuan@zaas.ac.cn (Y.C.); ruanmy@zaas.ac.cn (M.R.); yeqj@zaas.ac.cn (Q.Y.); wangrq@zaas.ac.cn (R.W.); zhougz@zaas.ac.cn (G.Z.); liu-l-jia@163.com (J.L.); 2 College of Horticulture and Gardening, Yangtze University, Jingzhou 434025, China; 3 Wulanchabu Academy of Agricultural and Forestry Sciences, Wulanchabu 012000, China; 4 College of Biotechnology, Jiangsu University of Science and Technology, Zhenjiang 212018, China; qdliuchaohi@163.com

**Keywords:** sucrose metabolism genes, polyploidization, *Solanum lycopersicum*, evolution, homologous relationship, expression pattern

## Abstract

Sucrose, the primary carbon transport mode and vital carbohydrate for higher plants, significantly impacts plant growth, development, yield, and quality formation. Its metabolism involves three key steps: synthesis, transport, and degradation. Two genome triplication events have occurred in Solanaceae, which have resulted in massive gene loss. In this study, a total of 48 and 65 genes from seven sucrose metabolism gene families in *Vitis vinifera* and *Solanum lycopersicum* were identified, respectively. The number of members comprising the different gene families varied widely. And there were significant variations in the pattern of gene duplication and loss in the tomato following two WGD events. Tandem duplication is a major factor in the expansion of the SWEET and Acid INV gene families. All the genes are irregularly distributed on the chromosomes, with the majority of the genes showing collinearity with the grape, particularly the CIN family. And the seven gene families were subjected to a purifying selection. The expression patterns of the different gene families exhibited notable variations. This study presents basic information about the sucrose metabolism genes in the tomato and grape, and paves the way for further investigations into the impact of SCT events on the phylogeny, gene retention duplication, and function of sucrose metabolism gene families in the tomato or Solanaceae, and the adaptive evolution of the tomato.

## 1. Introduction

Sucrose (Suc), the main end product of photosynthesis in photosynthetic bacteria and most higher plants, is translocated from the source leaves to sink tissues through phloem [1,2,3]. Sucrose metabolism plays critical roles in development, yield formation, and the stress response, primarily by producing a series of sugars as metabolites for fuel growth and the synthesis of indispensable compounds [4]. The sucrose metabolism process can be divided into three steps, including sucrose synthesis, sucrose transport, and sucrose degradation. Suc is synthesized by sucrose phosphate synthase (SPS) and sucrose phosphate phosphatase (SPP) in the cytosol. Uridine diphosphate glucose (UDPG) and fructose-6-phosphate synthesize sucrose-6-phosphate under the action of SPS [5]. SPP hydrolyzes Suc-6-phosphate to generate Suc. SPS is a key enzyme for sucrose synthesis in plants [4]. SPS activity has been correlated with plant growth, yield formation [6,7,8], and sucrose accumulation [9]. In addition, plants can promote their own growth and biomass accumulation through the interaction of SPS and SPP [10].

Sucrose could provide the carbon scaffolding and energy for plant growth and development. However, carbohydrate compounds cannot be transported independently across membranes, but require the assistance of sucrose transporters, such as sucrose transporters (SUT) and SWEET (Sugars will eventually be exported transporters). SUT can regulate the absorption efficiency of sucrose [11]. SUT genes play a crucial role in the transport of sucrose in the phloem of the potato [12], tomato [13], sweet orange [14], and rice [15]. Riesmeier cloned the SUT cDNA from spinach in 1992 [16]. The research on SWEET started relatively later than that on SUT. SWEET was first identified by Chen in 2010, and they reported on the function of the SWEET protein in *Arabidopsis* [17]. SWEET is a kind of protein that transports sugar on the cell membrane by using the pressure potential of the intracellular and extracellular sugar concentration gradient [18,19,20,21]. Moreover, studies have indicated that the SWEET gene family is involved in multiple functional regulations, such as sugar accumulation in fruits [14], reproductive development [19], and leaf senescence [22].

After being unloaded in the phloem, sucrose needs to be degraded into hexose for direct utilization by plant cells [23]. There are two enzymes involved in sucrose degradation in plants: invertase and sucrose synthase. Invertase (INV) hydrolyzes sucrose into fructose and glucose, and the process is irreversible. Sucrose synthase (SUS) can catalyze Suc and Uridine diphosphate (UDP) to produce UDPG and fructose, and the process is reversible [24]. INV can be classified into two main categories according to their optimum pH: the alkaline/neutral invertase (CIN), with an optimal pH between 7.0 and 7.8, and the Acid INV, with an optimal pH between 4.5 and 5.5 [25]. In addition, the acid invertases (Acid INV) are located in the cell walls or as a soluble form residing in the vacuoles, abbreviated, respectively, as cell wall invertase (CWIN) and vacuolar invertase (VIN). In addition to having a high degree of sequence similarity, CWINs and VINs also have a close evolutionary relationship. INV plays an important role in regulating sucrose metabolism, plant growth and development, and biotic and abiotic stress [26,27,28]. SUS is a biochemical marker in sink strength [23]. For example, restraining SUS expression leads to shrunken seeds in cotton [29] and maize [30], or to weakened starch accumulation in potato tubers [31]. Furthermore, the research indexed shows that SUS appears to be the main participant in the biosynthesis of lipids, differentiates, and carbohydrate compounds, like cellulose and starch [32]. Therefore, sucrose metabolism genes play a significant role in plant growth and development, yield formation, and stress responses. It would be valuable to obtain a better understanding of this large group of enzymes.

Ancient whole genome duplications (WGD) or polyploidizations (WGP) are wide-spread in plants. And they have always been regarded as important driving forces which help species to adapt the environment, to differentiate, and to evolve [33,34,35]. Many studies have shown that WGP is important for the complexity of the plant genome structure [36,37,38]. The occurrence of WGD leads to chromosome doubling, resulting in duplicated genes and alterations in the chromosome structure [39]. Duplicate genes are thought to be important for the generation of new functions and adaptive evolution [40,41]. Commonly selected gene duplicates from many lineages may play key roles in the process of responding to severe environmental stresses [40,42]. WGD also produces homologous fragments, which may lead to genomic instability, gene replacement, gene loss, or chromosome rearrangement [43]. The structural alterations of chromosomes may hold significant implications for their functional evolution. In addition, the duplicated genes generated by polyploidization can provide a large amount of material for the generation of new genes [44]. The evolutionary directions of duplicated genes include neo-functionalization, subfunctionalization, and non-functionalization [45,46]. Whole genome triplication is considered to have made significant contributions to the evolution of morphological and physiological diversity. It may be an important driver of genetic innovation [47,48].

The two major branches of angiosperms, pan-eudicots and monocots, separately experienced gamma (γ) events or tau (τ) events early in their evolutionary history [45,49]. After this, three rounds of WGDs (γ-β-α) occurred in the Arabidopsis thaliana lineage [50,51]. Similarly, the Solanum lineage underwent two whole-genome triplication (WGT) events: the first one was the ancient genome triplications and was shared with the rosids and asterids plants, and was known as the γ event [52]; the other was more recent (91-52 million years ago), and was accompanied by substantial gene loss [53,54]. However, the grape only experienced the first WGT, as it did not take part in the new, specific WGP event [55]. In this study, we focus on the more recent WGP event, which was regarded as a Solanaceae-common triplication (SCT) event. The tomato has plentiful nutrients and biological components for human health. It is versatile, and is capable of being consumed either fresh or processed, and can be used as both a fruit and vegetable [56,57]. It is widely cultivated around the world. The global yield was 189.03 million tons in 2021 (https://www.fao.org/faostat/en/#home (accessed on 1 December 2023)). The tomato is a member of the Solanaceae, which also includes crops such as potatoes and peppers. It is a highly worthy plant family for research [58]. In addition, the tomato serves as a model plant for studying the genetics, development, and physiological changes of fleshy fruits. It also has a relatively short growth cycle and is easy to cultivate [59]. The entire tomato genome has been sequenced, and high-quality genomes of the different tomato varieties have been obtained [60,61]. This provides more accurate and abundant information and resources for subsequent research, which can facilitate further gene function mining, evolutionary mechanism research, and applications [62]. Therefore, we chose the grape as a reference with which to study the changes to the sucrose metabolism genes in the tomato after this SCT event, so as to show the effect of SCT events on sucrose metabolism genes in Solanaceae.

WGDs have been associated with gene functional complexity, speciation, and organismal diversity [47]. There have been many studies on the genes associated with sucrose metabolism, but few have examined the divergent retention of sucrose metabolism genes from the perspective of WGD. In this study, utilizing the grape (*Vitis vinifera*) and Solanaceae model plant tomato (*Solanum lycopersicum*) as research subjects, a total of seven sucrose metabolism gene families were identified and characterized. A preliminary analysis of the evolutionary patterns of these gene families was conducted by examining their phylogenetic relationships, gene localization on chromosomes, synteny relationships, and Ka/Ks values. In addition, an evaluation was conducted on their expression profiles in several organs and tissues. The findings of this study offer a theoretical reference for further research on the adaptive evolution of sucrose metabolism genes in the tomato and their effects on tomato growth and development.

## 2. Results

### 2.1. Identification of Sucrose Metabolism Genes in Solanum Lycopersicum and Vitis vinifera

To identify the genes involved in sucrose metabolism in the tomato and grape, we constructed a local database and performed blastP searches using BioEdit v7.7 software (https://thalljiscience.github.io/page2.html, accessed on 1 September 2023). InterPro and HMMER were used to filter out the invalid and non-sucrose metabolism gene amino acid sequences from the retrieved data. In total, 113 sequences were identified. And a total of sixty-five genes related to sucrose metabolism were identified in *S. lycopersicum* and forty-eight in *V. vinifera* (Supplemental Table S1).

Among these seven kinds of gene families, SPS and SPPhad the same number of genes in the two species, the numbers were four and two (Supplemental Table S1). The others had changed to varying degrees. And a significant difference occurred in the SWEET and Acid INV, such that the amount was more in the tomato than in the grape. And the quantity of SWEETs and Acid INVs in *S. lycopersicum* was almost twice as much as in *V. vinifera*, while the remaining two gene families were slightly different.

### 2.2. Phylogenetic Analysis of Sucrose Metabolism Genes in Solanum lycopersicum and Vitis vinifera

**Figure 1:**
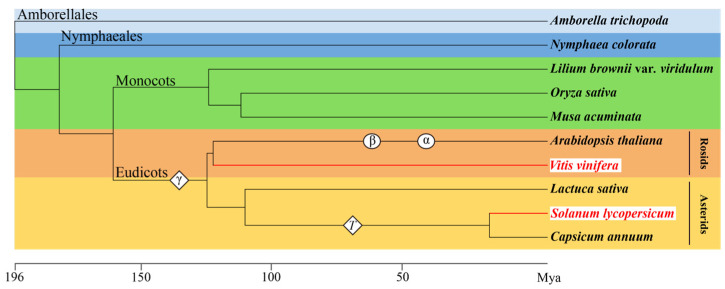
The phylogeny and timescales of 10 plant species. The time tree was constructed using the Timetree database and used the common order of angiosperms to show the evolutionary relationships of species. It includes the early divergent taxa of angiosperms, such as Amborellales (*Amborella trichopoda*) and Nymphaeales (*Nymphaea colorata*), as well as monocots (*Oryza sativa*, *Musa acuminata*, and *Lilium brownii* var. *viridulum*), and eudicots (*Arabidopsis thaliana*, *Vitis vinifera*, *Lactuca sativa*, *Solanum lycopersicum*, and *Capsicum annuum*). The WGP events described in previous studies [52,54,55,64] are mapped onto the tree. Mya (million years ago) represents the time of species differentiation.

The tomato and grape belong to the asterids and rosids, respectively. No new WGT event has occurred in the grape after the WGT-γ event shared by the eudicots [55]. In the Solanaceae species, 60–70 Mya years ago, another triplication event (WGT-T) took place. And this event was unique to the Solanaceae species [54,63] (Figure 1).

**Figure 2:**
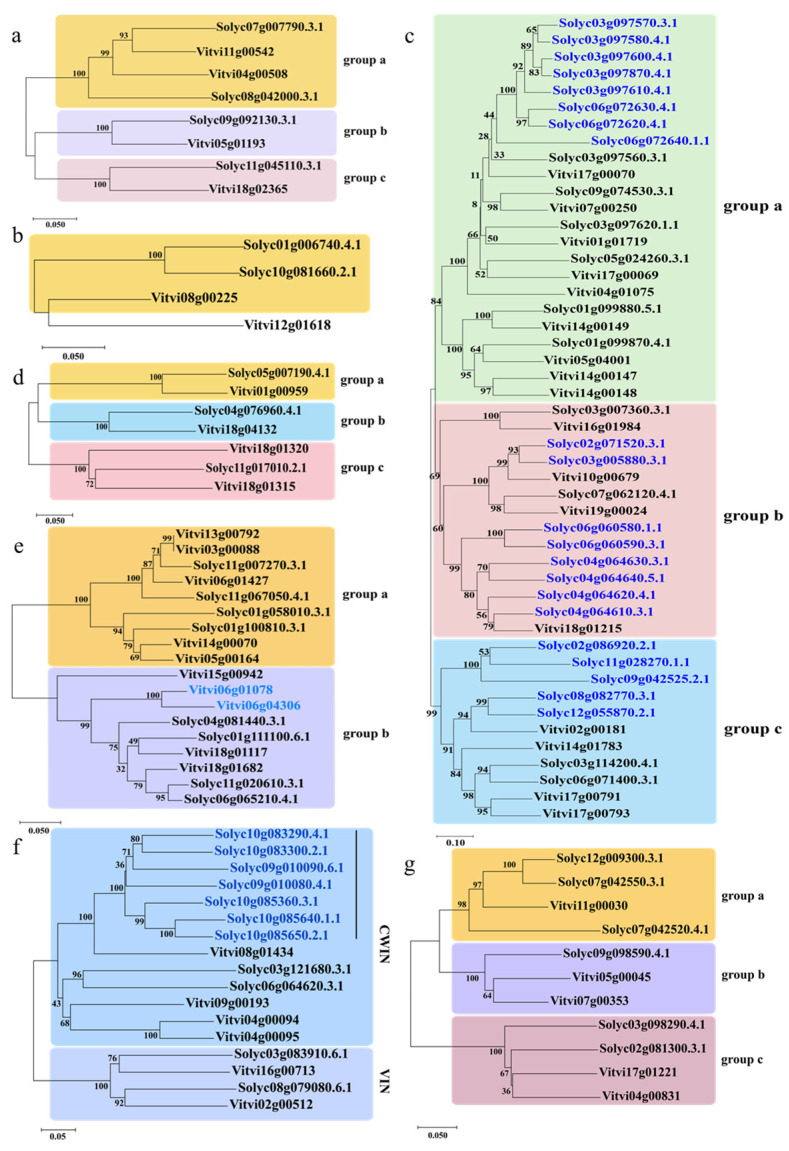
Phylogenetic relationships of sucrose metabolism gene families in the grape (*Vitis vinifera*) and tomato (*Solanum lycopersicum*). The phylogenetic trees were constructed using the neighbor joining (NJ) method and MEGA X software (https://www.megasoftware.net/dload_win_gui, accessed on 1 September 2023). The stability of the internal nodes was evaluated using 1000 bootstrap analyses, and the numerical values on the branches symbolize the bootstrap supports. (**a**) Sucrose phosphate synthase (SPS) gene family; (**b**) sucrose phosphate phosphatase (SPP) gene family; (**c**) SWEET gene family; (**d**) sucrose transporters (SUT); (**e**) alkaline/neutral invertase (CIN) gene family; (**f**) acid invertases (Acid INV) gene family; and (**g**) sucrose synthase (SUS) gene family.

To analyze the different evolutionary relationships of the plants Suc metabolism gene families, we further constructed NJ trees using the amino acids of 113 selected genes from *S. lycopersicum* and *V. vinifera* (Figure 2).

Considering the sucrose synthesis genes (Figure 2a,b), the results show that the SPSs after *S. lycopersicum* triplication did not have obvious gene insertion and loss, and it has only one copy retained (Figure 2a). The SPPs displayed different results, with the grape and tomato divided into two different branches. For the *Vitvi08g00225*, the tomato retained two copies (Figure 2b).

Considering the sucrose transport genes, there was a distinct difference in the number between the SUT and SWEET (Figure 2c,d). They both had duplicated and lost genes, which was more visible in the SWEETs. Based on the bootstrap values of the phylogenetic tree, the SWEETs were divided into three clades (Figure 2c). And in the three groups of blue words, the tomato genes were significantly doubled, which resulted from the presence of homologous repetitive genes. For the SUT gene, the tomato was extremely homologous to the grape, and retained only one copy after the SCT event.

Considering the sucrose degradation genes, the Acid INV retained more copy numbers than the CIN and the SUS in the tomato after SCT (Figure 2e–g). The Acid INV genes were divided into two clades. Clade 1 was CWINs, and clade 2 was INVs. They were obviously divided into two categories in the phylogenetic tree. This is consistent with the result of a previous study [65]. In addition, the CWIN genes in *S. lycopersicum* were clearly duplicated compared to those in *V. vinifera* (blue words). In contrast, VIN displayed a more conservative evolution after SCT [66]. The CINs were also divided into two groups. Group b was more conservative in its evolution than group a. The SUSs were divided into three groups. And in group a, the genome duplication event in *S. lycopersicum* was obviously revealed. Compared to the *V. vinifera* genes, the *S. lycopersicum* genes in group a showed replication.

### 2.3. Chromosomal Localization of Sucrose Metabolism Genes in S. lycopersicum and V. vinifera

**Figure 3:**
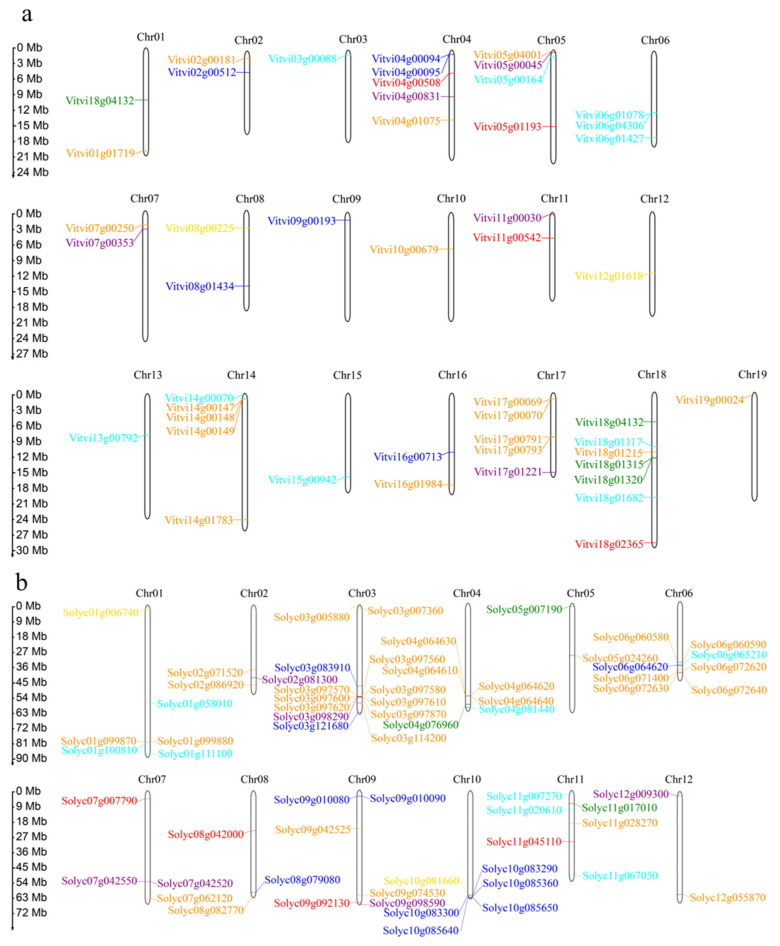
The chromosomal location of sucrose metabolism genes in *Vitis vinifera* (**a**) and *Solanum lycopersicum* (**b**). Different colors represent distinct sucrose metabolism gene families. Red, SPSs; gold, SPPs; dark orange, SWEETs; green, SUTs; cyan, CINs; blue, Acid INVs; purple, SUSs. Serial numbers are displayed atop each chromosome. The scale represents megabases (Mb), and the size of chromosomes is to be determined using the provided scale on the left.

The chromosomal location of all the sucrose metabolism genes in *S. lycopersicum* and *V. vinifera* were investigated on the basis of the physical position of the whole genes and are shown in (Figure 3). A total of 48 sucrose metabolism genes were unevenly distributed across the 19 chromosomes of the grape (Figure 3a). A grand total of 65 genes were accurately mapped onto 12 chromosomes of the tomato (Figure 3b). A significant disparity exists in the density of the sucrose metabolism genes on the different chromosomes. Furthermore, the majority of these genes were primarily distributed on the ends of the chromosomes. The chromosome Chr18 of *V. vinifera* carried the highest number of genes (7), and multiple chromosomes carried only one sucrose metabolism gene (Chr03/10/12/13/15/19). It is noteworthy that the sequence distances between the six gene clusters in the grape were less than 100 kb. These gene clusters belong to different gene families, such as the Acid INV (*Vitvi04g00094/95*), the CIN (*Vitvi06g01078* and *Vitvi06g04306*), the SWEET (*Vitvi14g00147/48*/*49*, *Vitvi17g00069*/70, and *Vitvi17g00791/93*), and the SUT (*Vitvi18g01315/20*), indicating tandem duplication events. In *S. lycopersicum*, chromosome Chr03 had the highest number of genes (13). Sequences with physical distances less than 100 kb were found in both the SWEET (*Solyc01g099870*/*80*, *Solyc03g097560/70/80*, *Solyc03g097600/10/20*, *Solyc04g064610/20/30/40*, *Solyc06g060580/90*, and *Solyc06g072620/30/40*) and Acid INV (*Solyc09g010080/90*, *Solyc10g083290, Solyc10g083300*, and *Solyc10g085640/50*) gene families. A previous study showed that six genes (*Solyc01g099870/80*, *Solyc04g064630/40*, and *Solyc06g060580/90*) within the SWEETs exhibited tight linkage [67], suggesting their classification as tandem duplication genes. Moreover, compared to *V. vinifera*, the number of tandem duplicated genes in the SWEETs had, significantly, doubled, whereas no tandemly duplicated genes were found in the SUTs in *S. lycopersicum*. Based on the chromosomal localization maps of *V. vinifera* and *S. lycopersicum*, it was observed that the genes involved in the sucrose synthesis pathway were scattered on different chromosomes without any quantitative alterations, suggesting a conservative distribution. In the sucrose transport pathway, the SWEETs were distributed on 11 different chromosomes. The SUT family was located on chromosome 01 and 18 in the grape, while in the tomato it was distributed across three different chromosomes. This distribution is likely attributed to the exchange and recombination of chromatin during the SCT event. Within the sucrose degradation pathway, only the CIN gene family, following SCT, had a lower number of genes in the tomato compared to the grape. Additionally, the chromosomes carrying the CIN gene also underwent a reduction in the tomato. The number of chromosomes was conserved between the Acid INV and SUS gene families.

### 2.4. Homology Relationship and Synteny Analysis

**Figure 4:**
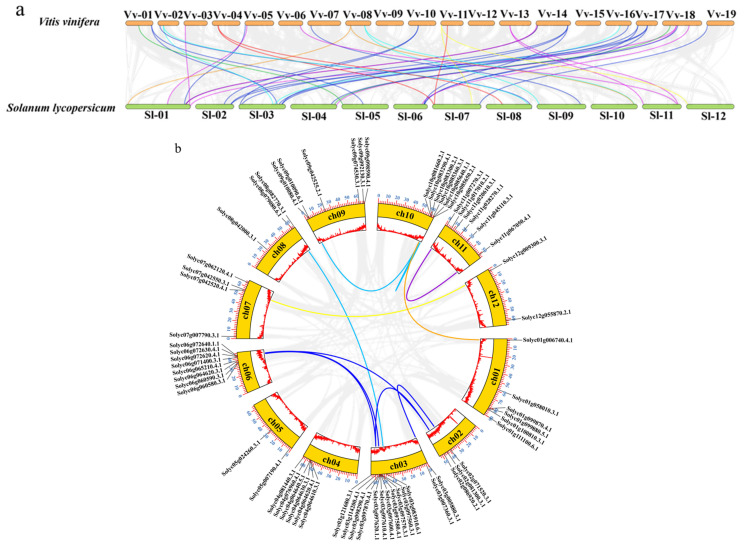
The homology analysis. (**a**) Synteny analysis of sucrose metabolism genes in *V. vinifera* and *S. lycopersicum*. The number of chromosomes is displayed at the top of each chromosome, and the lengths of the orange and green boxes represent the lengths of grape and tomato chromosomes. (**b**) Synteny analysis of sucrose metabolism genes in *S. lycopersicum*. The lengths of the yellow boxes represent the sizes of the tomato chromosomes, and they are labeled with blue numbers on the outer side indicating megabases (Mb). The lengths of the red line segments inside the white boxes represent the gene density on different chromosomes. The number of chromosomes is displayed within the yellow box. For (**a**,**b**), the gray lines in the background exhibit the homologous relationship, and different color lines represent distinct gene families. Red represents SPSs, orange represents SPPs, blue represents SWEETs, green represents SUTs, purple represents CINs, cyan represents Acid INVs, and yellow represents SUSs.

The homologous genes can be divided into orthologous and paralogous genes. We used TBtools v2.027 software (https://www.yuque.com/cjchen/hirv8i/xq65ml, accessed on 1 December 2023) to predict the homology relationship of the sucrose metabolism genes between *S. lycopersicum* and itself and *V. vinifera*, and the syntenic relationship was visualized. The homology analysis of the sucrose metabolism gene families in *S. lycopersicum* and *V. vinifera* showed that there were homologous relationships among them (Figure 4a). A total of 34 genes in *S. lycopersicum* showed synteny with *V. vinifera*. Among the synteny genes, *Solyc07g007790* and *Solyc08g042000* had syntenic relationships with *Vitvi04g00508* in the SPS gene family. Similarly, *Solyc01g006740* and *Solyc10g081660* had syntenic relationships with *Vitvi08g00225* in the SPPs. For the SWEETs, only 15 members displayed homology relationships with the grape genomes. In the SUTs, except for *Solyc11g017010*, the other two genes were homologous. For the sucrose degradation genes, there were more homologous genes in the CINs than in the Acid INV and SUS in the *S. lycopersicum* and in the *V. vinifera* sucrose metabolism genes. Among the Acid INVs, only four members had synteny genes. And in the SUSs, two members were homologous with one gene (*Solyc07g042520*) in *V. vinifera*. In addition, from Figure 4a, it can be observed that the genes with collinearity between the grape and tomato are unevenly distributed on the chromosomes, and they do not have a one-to-one correspondence. This indicates that in the tomato, in addition to gene duplication and loss events following the SCT event, chromosomal rearrangements may have also occurred.

In addition, we also used TBtools to analyze the paralogous relationship of the sugar metabolism-related genes in the tomato, and the results are displayed in a circle diagram (Figure 4b). A homology analysis of these genes in *S. lycopersicum* indicated that there were syntenic paralogs relationships in the tomato. However, not all the families were homologous. Most of the sucrose metabolism genes in the tomato retained only one copy after the SCT event, with a greater occurrence of gene loss than gene duplication events. Duplication retention events were more obvious in the SWEETs. The SPS and SUT did not have paralogous genes. The SPP had two members, the SWEET had ten members, the CIN had two members, the Acid INV had eight members, and the SUS had two members.

In order to appreciate whether natural selection played a role in the evolution of the seven families related to sucrose metabolism in *S. lycopersicum*, a selection pressure analysis was conducted on the syntenic sucrose metabolism gene pairs between *S. lycopersicum* and *V. vinifera*. The Ka, Ks, and Ka/Ks ratio values were calculated. The calculation results are shown in Table 1. The results show that all the Ka/Ks ratios were less 1, which indicates that these genes were subjected to purifying selection pressure. Furthermore, eight pairs of genes had a Ka/Ks value less than 0.1, which may indicate a stronger purifying selection stress. This may choose to eliminate deleterious mutations, leaving the protein as it is. The purifying selection was the dominant force driving the evolution of the seven sucrose metabolism-related genes between the tomato and grape (Table 1).

Then, we calculated the Ka/Ks values of the sucrose metabolism genes (SPP, SWEET, CIN, Acid INV, and SUS) with syntenic relationship in *S. lycopersicum* (Table 2). The Ka/Ks ratios of these gene pairs were also less than 1. The results suggest that these genes seemed to evolve under purifying selection.

### 2.5. Expression Patterns of Sucrose Metabolism Genes in Different Tissues of S. lycopersicum

**Figure 5:**
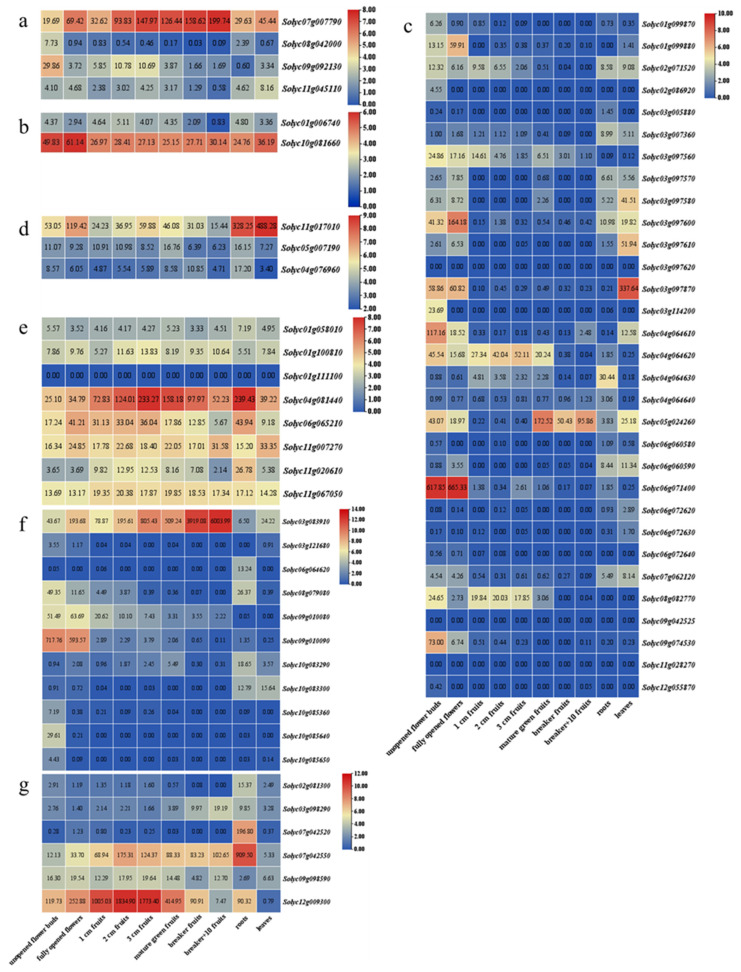
Expression patterns of seven sucrose metabolism gene families in different tissues of *S. lycopersicum*. (**a**) SPS gene family; (**b**) SPP gene family; (**c**) SWEET gene family; (**d**) SUT gene family; (**e**) CIN gene family; (**f**) Acid INV gene family; (**g**) SUS gene family. The color scale represents log_2_ transformed counts normalized using RPKM, where blue denotes low relative abundance and red signifies high relative abundance. (RPKM: reads per kilobase of exon per million reads mapped.)

The expression levels of the SPP, SPS, SUT, SWEET, SUS, and INV genes in five tissues/organs of the tomato (Heinz1706) were explored, which included the bud, flower, fruit, root, and leaf. The results show that these genes were expressed with varying expression patterns in the different organs and developmental stages (Figure 5).

For the genes involved in sucrose synthesis (Figure 5a,b), both the SPS (Figure 5a) and SPP (Figure 5b) had a gene with a relatively high expression level in all the investigated organs, which were *Solyc07g007790* and *Solyc10g081660*. In addition, *Solyc07g007790* was most highly expressed in the flowers, and *Solyc10g081660* was most highly expressed in the breaker fruits. For the genes involved in sucrose transport (Figure 5c,d), the expression levels were unbalanced. With the exception of *Solyc11g017010*, the other SUT genes were expressed at low levels in roots, leaves, flowers, and fruits. *Solyc11g017010* was expressed in the different organs and stages, with higher expression levels found in the roots and leaves. The expression patterns of the SWEETs were different from those of the SUTs, while *Solyc06g071400* was more highly expressed selectively in specific tissues (unopened flower buds and fully opened flowers). Apart from that, only five genes (*Solyc03g097600*, *Solyc03g097870*, *Solyc04g064610*, *Solyc05g024260*, and *Solyc09g074530*) were selectively highly expressed in specific organs and stages, eleven were hardly expressed, and the remaining SWEETs were weakly expressed.

For the genes involved in sucrose degradation (Figure 5e–g), all had a gene with a higher transcript level in the fruit, which were *Solyc04g081440*, *Solyc03g083910*, and *Solyc12g009300*. The results for the CINs showed that they were all expressed in the tested tissues except for *Solyc01g111100*, which was not expressed at all, and *Solyc04g081440*, which was expressed at constitutively higher levels in each tissue, especially in the roots and 3 cm fruits. The expression pattern of the Acid INV genes were specifically expressed. *Solyc03g083910* was expressed at a significantly high level in specific tissues and gradually increased along with the growth and development of the fruit. *Solyc09g010090* was expressed at a primarily high level in the unopened flower buds and fully opened flowers. The remaining Acid INV genes were either not expressed or poorly expressed. The transcript profiles of the SUSs were altered in the various organs. *Solyc07g042550* and *Solyc07g042520* had a higher expression in the roots. Spectacularly, the expression of levels of the *Solyc12g009300* gene was considerably higher in the unripe fruits (1 cm, 2 cm, and 3 cm fruits), and it was down-regulated with the development of fruit.

## 3. Discussion

Sucrose metabolism is important for plant growth and development, yield formation, and resistance to stress [4]. Studies on the gene families related to sucrose metabolism have been carried out for *Arabidopsis* [68], muskmelon [69], grape [70], pear [71], sugarcane [72], rice [15] and sweet potato [73]. In this study, we systematically identified, classified, evolved, and expressed these sucrose metabolism genes in the tomato (*S. lycopersicum*). In total, we identified 4, 2, 31, 3, 8, 11, and 6 genes of SPS, SPP, SWEET, SUT, CIN, Acid INV, and SUS in *S. lycopersicum*, and 4, 2, 17, 4, 10, 6, and 5 genes of SPS, SPP, SWEET, SUT, CIN, Acid INV and SUS in *V. vinifera*. Interestingly, the same number of SPS and SPP family members was observed in the tomato and grape. Combining the phylogenetic tree and syntenic relationship, we found that the relationship between the tomato and grape genes in the two gene families did not have a one-to-one correspondence. This may be due to the duplication and loss of these genes in the process of species evolution. But they were conserved in quantity and the gene families were small. Gene duplication and loss (segmental and tandem) is considered one of the basic driving forces of plant gene family expansion, which is common in the process of species evolution [74,75]. In addition, we found that the SWEET, SUT, CIN, Acid INV, and SUS had different numbers in the tomato and grape. This may be due to apparent replication and retention after the SCT event in the tomato.

Polyploidization is an important reason for the complexity of plant genome structures [34,50]. And the most important product of polyploidization events for the genome of a species is the large number of repetitive genes, which lead to plant divergence. But after polyploidization, many duplicated genes are lost during species evolution [48,64,76]. In addition, rearrangements, gene loss, gene inversions, and other developments are prone to occur after genome duplication [45,55]. Comparative genomic research has shown that the Solanaceae species experienced triploidy at the genomic level about 64–75 million years ago [77]. In Solanaceae, capsicum first diverged 29 Mya, and the tomato diverged relatively recently, about 12 Mya [63]. And the divergence of the tomato and pepper was considered to be a triplication, accompanied by the loss of a large number of genes [54]. In this study, nearly 52% of the sucrose metabolism genes in *S. lycopersicum* were syntenic with *V. vinifera*, which suggests that the SCT event might have contributed to the expansion of the sucrose metabolism genes in *S. lycopersicum*. Tandem duplication is one of the main ways to expand a gene family [78]. Tandem duplicate genes are defined as at least two homologous genes with a physical distance of less than 100 kb. Studies have shown that the Suc metabolic families containing tandem duplicated genes in the grape are the Acid INVs, CINs, SWEETs, and SUTs, which are marked on chromosomes Chr01, Chr04, Chr06, Chr14, Chr17, and Chr18. But in *S. lycopersicum*, tandem duplicated genes were found only in the SWEET and Acid INV, and they were distributed on six chromosomes, including Chr01, Chr03, Chr04, Chr06, Chr09, and Chr10. This result suggests that not all sucrose metabolism families expanded their family members by tandem duplication. After the Solanaceae genome triplication, the numbers of the SWEETs and Acid INVs were markedly doubled, and tandem duplication was a factor for the two family’s expansions in *S. lycopersicum*. However, after genome polyploidization, not all the duplicated genes were retained [79]. Moreover, after the tomato experienced the SCT event, chromosomal rearrangements may have occurred. Sucrose metabolism genes are irregularly distributed at the ends of chromosomes. Except for the CIN family in *S. lycopersicum*, members of the other sugar metabolism families increased after the SCT event. We appraised 10 members in *V. vinifera* and 8 members in *S. lycopersicum*. The relationship between the phylogenetic tree and the syntenic showed that the tomato’s CIN genes did not completely correspond to the grape’s CIN genes. There was no syntenic relationship among the four genes in *V. vinifera*, including *Vitvi03g0088*, *Vitvi15g00942*, *Vitvi06g01078*, and *Vitvi06g04306*. *Vitvi06g01078* and *Vitvi06g04306* were clustered into one group, and they were tandem repeat genes. We speculate that genes may have been lost after the SCT event in *S. lycopersicum*.

The nonsynonymous/synonymous ratio of sucrose metabolism genes between *S. lycopersicum* and *V. vinifera* indicate that homologous genes were not positively selected in the process of evolution. The seven gene families, including SPS, SPP, SWEET, SUT, CIN, Acid INV, and SUS, were subjected to purifying selection pressure. And functions may be well-maintained during evolution. This is consistent with previous studies. The expansion of the SWEET genes in Solanaceae under purifying selection was mainly due to tandem duplication [20]. Similarly, in our paper, the SWEET gene family had more tandem duplicated genes in the tomato than in the grape. And it was the main reason for the increase in SWEET members in the tomato after the SCT event. The Acid INV genes had come under a relaxed purifying selection pressure, but the CINs evolved under a greater purifying selection than that of the Acid INVs [65]. The overall Ka/Ks value of the CINs is lower than that of the CWINs and VINs. The number of tandem duplication genes in Acid INV was also significantly increased, which may be the product of the SCT event. The SUS genes were divided into three subfamilies in angiosperms. And SUS III evolved under a relaxed purifying selection, while SUS I evolved under stronger evolutionary constraints [53]. Our results support the point that the SUS were divided into three groups, and group a (*Solyc12g009300* and *Solyc07g042520*) experienced a stronger purifying selection.

SPS is a vital regulator of sucrose synthesis, which regulates sucrose content in higher plants. Sucrose content and biomass production in sugarcane could be increased by the overexpression of SPS [72]. *Solyc07g007790* in the SPS was expressed in the flower buds, flowers, fruit, roots and leaves, and especially in the fruit. On the contrary, *Solyc09g092130* has a relatively high expression in the flower buds. This situation indicates that SPSs may play an important role in tomato fruit ripening or sugar accumulation. SPP can affect the distribution of carbon in carbohydrate partitioning, regulate the synthesis of sucrose and affect plant growth [80]. In the SPPs, only *Solyc01g006740* performed a function in all the selected materials, while *Solyc10g081660* was basically not expressed. There are many members of the SWEET protein family, and different SWEET members may have different functions. It has been proven that it plays an important role in participating in sugar transport [81]; affects the growth and development of plant roots, stems, and leaves [18,22]; participates in the growth and development of flowers, fruits, and seeds [82,83]; and responding to stress [84,85]. In our study, 11 SWEETs had lower expression levels or no expression in these organs, including the buds, flowers, fruits, roots, and leaves. *Solyc06g071400* was specifically highly expressed in unopened flower buds and fully opened flowers. *Solyc03g097870* was mainly expressed in leaves, with little expression in flower buds, flowers, and breaker fruits. *Solyc06g071400*/*Lestd1* was specifically expressed in mature pollen grains [86]. *Solyc09g074530* was specifically expressed in flower buds. In general, duplicate genes could be retained not only through subfunctionalization, where the duplicate genes perform different aspects of the original gene’s function, but also through neo-functionalization, where one of the genes acquires a new function; in addition, it may facilitate adaptation to varying environments [87,88]. Both *Solyc04g064610* and *Solyc04g064620* (one pair of paralogous genes) were expressed in the flower buds, flowers, and leaves, while *Solyc04g064620* were extra expressed in the tomato fruits at various stages. The other four pairs of paralogous genes (*Solyc01g099870*/*80*, *Solyc03g097560*/*70*, *Solyc03g097600*/*10*/*20*, and *Solyc04g064630*/*40*) showed similar conditions. These results indicate that the SWEET gene family shows differential expression patterns after duplication through transforming expression patterns in the development of plants and adapting to environmental change. SUTs were widely found in the tissues and cells of higher plants, and play vital roles in phloem loading, apoplast pathway transportation, and sink organs development [13,89]. And Dicotyledonous plants have only three subfamilies: SUT1, SUT2, and SUT4 [89]. In *S. lycopersicum*, *Solyc11g017010* (*SlSUT1*) was expressed in selected organs, especially in the leaves and roots. *SlSUT1* may have a great effect on the development of the plant source and sink organs. Inhibiting the expression of *SlSUT1* in the tomato caused leaf curling and wilting, premature senescence, and affected the sink organs’ development [13].

SUS can provide the substrate UDPG or the indirect substrate adenosine diphosphate glucose (ADPG) for polysaccharides, such as cellulose and starch, so it plays an important role in plant carbon distribution [90,91,92]. *Solyc12g009300* was highly expressed in the fruit growth and development stage. *Solyc07g042550* had high transcript level in the roots, while *Solyc07g042520* was only expressed in the roots. In agreement with this, the activity of the SUS was mostly higher in the developing fruits and seeds than in the leaves [93]. These facts suggest that SUS plays an important role in sink development. For invertase, which also hydrolyzes sucrose, six out of the eight CIN genes were broadly or constitutively expressed in all the tissues and developmental stages, while the two other CIN genes were hardly expressed. What is interesting is that the Acid INV exhibited opposite expression profiles; nine out of the eleven Acid INV genes were weakly expressed or not expressed, while *Solyc03g083910* (VIN) was expressed at a significantly high level in floral development and in most of the tissues, especially in the breaker fruit. *Solyc08g079080* (VIN) was expressed in the buds and roots, which may be a functional supplement to *Solyc03g083910*. *Solyc09g010090* was highly transcripted in the buds and flowers. In brief, the CINs showed broader expression patterns than the Acid INV. CINs are evolutionarily and functionally more conserved than Acid INV, which was discovered by comparing the phylogenetic and functional genomic analyses [65]. Compared to the genes expressed in specific tissues or stages, the growth and development of plants is more likely to be impacted by the broadly expressed genes [94]. Furthermore, not all tandem duplicated genes were expressed in these seven families; for example, *Solyc06g072630*/*40* were not expressed at all stages in the selected material. A possible explanation is that gene duplication and divergence play crucial roles in the evolution of species, which can provide the raw genetic material for biological evolution [33]. A large number of duplicated genes were found in the genome that greatly promoted the evolution of the structure and function of the genome. The duplicated genes may experience deletion, hypofunctionalization, neofunctionalization, among others [43,87,88,95,96]. In conclusion, the seven gene families exhibited different expression patterns during the stages of tomato development, and for the genes which were preferentially retained (SWEET and Acid INV genes), not all displayed high expression levels. This suggests that the tomato’s sucrose metabolism requires the cooperative action of numerous enzymes.

Overall, this study indicates that the tomato’s sucrose metabolism genes underwent gene duplication, retention, and loss after the SCT event. This could be attributed to the importance of sucrose metabolism for plant growth and development, and that these genes were subjected to a strong purifying selection. Additionally, chromosomal rearrangements may have occurred during the triplication process. The different gene families involved in sucrose metabolism exhibit different expression patterns due to their diverse functions in plants. This study systematically analyzed the sucrose metabolism genes in the tomato and grape, the latter of which can provide a reference for the study of the SCT event on tomato growth and development, as well as the adaptive evolution of the sucrose metabolism genes. However, this study only used two representative species, the grape and tomato, as research subjects, and did not analyze the sucrose metabolism gene families of the entire Solanaceae crop. Moreover, the tomato and grape belong to different evolutionary lineages, and the grape may have evolved its own unique sucrose metabolism genes in the course of evolution to aid its own growth and developmental needs. By adding one or more additional reference species, it would be possible to better investigate the selection patterns of the sucrose metabolism genes in the Solanaceae, and their impact on the growth and development of Solanaceae crops.

## 4. Materials and Methods

### 4.1. Identification of Sucrose Metabolism Genes in Solanum lycopersicum and Vitis vinifera

Information about the whole genome and the gene annotation data for *Solanum lycopersicum* were downloaded from Sol Genomics Network (https://solgenomics.net/ (accessed on 1 September 2023)), and the versions were SL4.0 and ITAG4.1. Information about the whole genome and gene annotation data for *Vitis vinifera* were downloaded from the EnsemblPlants (http://plants.ensembl.org/species.html (accessed on 1 September 2023)) database, and the version was PN40024.v4. After that, a local database was created. BioEdit v7.7 software (https://thalljiscience.github.io/page2.html, accessed on 1 September 2023) was used for managing the locally downloaded data. The Blastp algorithm in the BioEdit software was used to search for the protein sequences of all the sucrose metabolism gene families in the tomato and grape, and the E-value was set to 10^−6^. All sequences were summarized, and the structural domains and the Hidden Markov Model (HMM) profile of all amino acid sequences were analyzed using InterPro (https://www.ebi.ac.uk/interpro/ (accessed on 1 September 2023)) and HMMER (https://www.ebi.ac.uk/Tools/hmmer/search/hmmscan (accessed on 1 September 2023)). The HMM profile of Sucrose_synth (PF00862), Glycos_transf_1 (PF00534), and S6PP (PF05116) were the typical conservation domains of the SPSs; the SPPs had S6PP (PF05116) and S6PP_C (PF08472) domains. The SUTs belong to the major facilitator superfamily (MFS), and its typical conservation domain was MFS_2 (PF13347); the exemplary conserved domain of the SWEETs was MtN3_slv (PF03083). The Glyco_hydro_100 (PF12899) was the domain of the CINs, and Glyco_hydro_32N (PF00251) and Glyco_hydro_32C domain (PF08244) were the classic conserved domains of the Acid INV. The SUSs owned two conservation domains, which were Sucrose_synth (PF00862) and Glycos_transf_1 (PF00534). All the protein sequences containing the representative domains were derived as candidates. However, some sequences obtained through Blast had the deletion of a sequence or conserved domains, which were probably due to genome annotation errors. For the problematical gene sequences, we used the database for the corresponding genomic sequences and reannotated those genes using the online website Softberry (http://www.softberry.com/berry.phtml? (accessed on 1 September 2023)). In this way, the sucrose metabolism genes, including the SPPs, SPSs, SUTs, SWEETs, SUSs, CINs, and Acid INVs genes, were acquired.

The tomato reference genome used in this study is Heinz1706 [60,62], which is a modern cultivated variety. The *Solanum lycopersicum* cv. Heinz 1706 genome sketch has been widely used as a reference genome for scientific research since its release in 2012 [54]. The genome assembly version used in this paper is SL4.0, and the gene annotation version is ITAG4.1 (https://solgenomics.net/ftp/tomato_genome/Heinz1706/ (accessed on 1 September 2023)). PN40024 was formed through 9 selfings of cv. “Helfensteiner” (cross of cv. “Pinot noir” and “Schiava grossa”), rather than being a single “Pinot noir” [97]. It is a highly homozygous cultivar that serves as the reference genome for the grape and was first sequenced in 2007 [55]. The genome assembly and gene annotation version utilized in this study is PN40024.v4 (https://plants.ensembl.org/Vitis_vinifera/Info/Index (accessed on 1 September 2023)).

### 4.2. Phylogenetic Analysis

To display the evolutionary relationships between the tomato, grape, and other species, we selected several angiosperms, including representatives of lineages from the earliest diverging lineages within extant mesangiosperms (the ANA-grade groups), like *Amborella trichopoda* in the order Amborellales, and *Nymphaea colorata* in the order Nymphaeales. In addition, there are common monocot plants, such as *Oryza sativa*, *Musa acuminata*, and *Lilium brownii* var. *viridulum*, as well as eudicot plants, including *Amborella trichopoda* and *Vitis vinifera* from the order Rosids, and *Lactuca sativa*, *Solanum lycopersicum*, and *Capsicum annuum* from the order Asterids. TimeTree (http://www.timetree.org/ (accessed on 1 September 2023)) was used to construct the phylogenetic trees of species. The WGT events that have occurred are marked on the phylogenetic tree with reference to existing studies [48,53,54,63,98].

And then, to study the screening of sucrose metabolism genes in the tomato by the SCT event, we used the seven gene families identified in *S. lycopersicum* and *V. vinifera* to construct phylogenetic trees. The phylogenetic trees of the gene families (SPP, SPS, SUT, SWEET, Acid INV, CIN, and SUS) were constructed by using the neighbor-joining (NJ) method, with 1000 bootstrap replicates in MEGA X software (https://www.megasoftware.net/dload_win_gui, accessed on 1 September 2023) [99]. Then, ITOL was used to beautify the phylogenetic trees (https://itol.embl.de/itol.cgi (accessed on 1 September 2023)). Phylogenetic groups were classified based on the bootstrap values.

### 4.3. Chromosomal Localization and Synteny Analysis

Chromosomal position mapping of sucrose metabolism genes in tomato was performed using MG2C (http://mg2c.iask.in/mg2c_v2.1/ (accessed on 1 September 2023)) [100]. If two genes belong to the same gene family and the physical distance between them is less than 100 kb, they are defined as tandem duplicated genes [101]. An analysis and visualization of collinearity between *S. lycopersicum* and itself, and *V. vinifera* was accomplished using TBtools [102]. To explore the pattern of selection (purifying selection, positive selection, or neutral selection) among the paralogous genes in tomato (*S. lycopersicum*), the ratios of the nonsynonymous substitution rate (Ka) to the synonymous substitution rate (Ks) were calculated using TBtools v2.027 software (https://www.yuque.com/cjchen/hirv8i/xq65ml, accessed on 1 December 2023), based on the CDS sequences. In addition, a Ka/Ks ratio less than 1 represents purifying selection, otherwise it is positive or neutral selection [103].

### 4.4. Transcription Expression Pattern Analysis

To analyze the transcript profile of the genes involved in sucrose metabolism in tomato’s (*S. lycopersicum*) various tissues/organs, the Tomato Functional Genomic Database (TFGD) (http://ted.bti.cornell.edu/ (accessed on 1 September 2023)) was utilized. Eventually, four tissues from Heinz (*S. lycopersicum*) were selected, including the flower, fruit, root, and leaf. The flower samples were divided into unopened flower buds and fully opened flowers. Fruits were collected by size. Gene expression profiles were constructed using TBtools for further analysis. And the transcript profiles were generated using log_2_ (RPKM+1) values of these genes.

## 5. Conclusions

WGDs contribute to genome novelty by preserving a portion of gene duplicates, and it is also related to speciation, adaptability, and diversity. The tomato is one of the most important crops in the Solanaceae, and is a classic model plant. In this study, seven sucrose metabolism gene families from the tomato and grape were systematically analyzed for their phylogeny, chromosomal organization, evolutionary mechanisms, divergent retention of genes, and expression patterns. The basic bioinformatics analysis of the tomato sucrose metabolism genes in this study provides fundamental resources for the further excavation of the retention mode, evolutionary patterns, and functional studies of the sucrose metabolism genes in the tomato or Solanaceae after the SCT event.

## Figures and Tables

**Table 1:** The nonsynonymous (Ka), synonymous (Ks), and Ka/Ks ratio of syntenic gene pairs in *S. lycopersicum* and *V. vinifera*. Ks and Ka were calculated using TBtools.

	*Solanum lycopersicum*	*Vitis vinifera*	Ka	Ks	Ka/Ks
SPS	*Solyc07g007790*	*Vitvi11g00542*	0.11362809	1.33703922	0.08498486
	*Solyc07g007790*	*Vitvi04g00508*	0.17194748	1.39563813	0.123203481
	*Solyc08g042000*	*Vitvi04g00508*	0.19964061	1.04116391	0.19174753
SPP	*Solyc01g006740*	*Vitvi08g00225*	0.16184532	1.33939481	0.120834664
	*Solyc10g081660*	*Vitvi08g00225*	0.16741032	1.06108889	0.157772192
SWEET	*Solyc02g086920*	*Vitvi14g01783*	0.40602836	1.40050661	0.289915347
	*Solyc02g086920*	*Vitvi17g00791*	0.41698207	2.39161681	0.174351537
	*Solyc01g099870*	*Vitvi14g00147*	0.35410311	1.36055722	0.260263296
	*Solyc01g099870*	*Vitvi05g04001*	0.2686224	1.48840512	0.180476669
	*Solyc01g099880*	*Vitvi14g04044*	0.66660488	N	N
	*Solyc02g071520*	*Vitvi10g00679*	0.21747408	1.14595337	0.189775683
	*Solyc03g005880*	*Vitvi10g00679*	0.19707583	1.95513806	0.10079893
	*Solyc03g007360*	*Vitvi16g01984*	0.21263783	2.28626265	0.093006738
	*Solyc03g097560*	*Vitvi01g01719*	0.32878582	N	N
	*Solyc03g097560*	*Vitvi17g00069*	0.37389825	2.41720521	0.154682045
	*Solyc03g114200*	*Vitvi14g01783*	0.39521583	N	N
	*Solyc03g114200*	*Vitvi17g00791*	0.26071019	1.43942492	0.181121076
	*Solyc04g064610*	*Vitvi18g01215*	0.17285757	2.93820554	0.058831
	*Solyc05g024260*	*Vitvi01g01719*	0.36347455	2.05818095	0.176599899
	*Solyc06g071400*	*Vitvi14g01783*	0.36411637	3.26503459	0.111519912
	*Solyc06g071400*	*Vitvi17g00791*	0.23992208	3.88509738	0.061754458
	*Solyc07g062120*	*Vitvi19g00024*	0.24415894	1.29049766	0.189197504
	*Solyc08g082770*	*Vitvi02g00181*	0.3428003	2.44187365	0.140384128
	*Solyc09g074530*	*Vitvi07g00250*	0.26903608	N	N
	*Solyc06g072620*	*Vitvi17g00069*	0.30971683	1.9635318	0.157734566
SUT	*Solyc05g007190*	*Vitvi01g00959*	0.16589023	1.15491739	0.143638179
	*Solyc04g076960*	*Vitvi18g04132*	0.16269373	1.61605975	0.10067309
CIN	*Solyc01g100810*	*Vitvi14g00070*	0.14473684	1.32718543	0.10905548
	*Solyc01g111100*	*Vitvi18g01117*	0.1469987	1.71248171	0.085839576
	*Solyc01g111100*	*Vitvi03g00088*	0.11761239	1.2100907	0.097193039
	*Solyc04g081440*	*Vitvi18g01117*	0.12531074	1.23472624	0.101488682
	*Solyc11g007270*	*Vitvi13g00792*	0.13692573	1.09844259	0.124654428
	*Solyc11g067050*	*Vitvi13g00792*	0.16899417	1.54111736	0.109656911
	*Solyc11g067050*	*Vitvi06g01427*	0.18299346	1.20043492	0.152439299
	*Solyc06g065210*	*Vitvi18g01682*	0.06598712	1.43375694	0.046023927
	*Solyc01g058010*	*Vitvi05g00164*	0.17749537	1.58143294	0.112237049
CWIN	*Solyc09g010080*	*Vitvi08g01434*	0.25575845	1.64857908	0.155138722
	*Solyc10g083290*	*Vitvi08g01434*	0.23448308	1.73611241	0.135062155
VIN	*Solyc03g083910*	*Vitvi16g00713*	0.267711178	1.948821335	0.137370816
	*Solyc03g083910*	*Vitvi02g00512*	0.276151171	2.657257382	0.103923381
	*Solyc08g079080*	*Vitvi02g00512*	0.29190687	2.080093594	0.140333527
SUS	*Solyc12g009300*	*Vitvi11g00030*	0.10168938	1.58385903	0.064203557
	*Solyc07g042520*	*Vitvi11g00030*	0.17376854	1.49401818	0.116309523

**Table 2:** The nonsynonymous (Ka), synonymous (Ks) and Ka/Ks ratio of syntenic gene pairs in *S. lycopersicum*. Ks and Ka were calculated using TBtools.

	*Solanum lycopersicum*	*Solanum lycopersicum*	Ka	Ks	Ka/Ks
SPS	*Solyc01g006740*	*Solyc10g081660*	0.062151704	0.65616436	0.094719719
SWEET	*Solyc02g086920*	*Solyc03g114200*	0.466864795	3.05815308	0.152662336
	*Solyc02g086920*	*Solyc06g071400*	0.485013419	3.28256218	0.147754526
	*Solyc02g071520*	*Solyc03g005880*	0.108893908	0.89339854	0.121887269
	*Solyc03g097610*	*Solyc06g072630*	0.201591662	0.89821045	0.224437004
	*Solyc02g086920*	*Solyc03g114200*	0.466864795	3.05815308	0.152662336
	*Solyc03g114200*	*Solyc06g071400*	0.205827634	1.19510213	0.172225979
CIN	*Solyc11g007270*	*Solyc11g067050*	0.201332217	1.87098003	0.107607892
Acid INV	*Solyc09g010080*	*Solyc10g083290*	0.158937563	1.07235908	0.148213006
	*Solyc10g083290*	*Solyc10g085650*	0.183792572	1.14966465	0.159866246
	*Solyc10g083300*	*Solyc10g085640*	0.203293349	1.55918308	0.130384527
	*Solyc03g083910*	*Solyc08g079080*	0.308055691	2.07739243	0.148289599
SUS	*Solyc07g042520*	*Solyc12g009300*	0.193685014	2.37941318	0.081400328

## Data Availability

Data is contained within the article and Supplementary Material.

## Supplementary Materials

The following supporting information can be downloaded at: https://www.mdpi.com/article/10.3390/plants12244145/s1, Supplementary Tables S1 and S2, Details of the number and sequences of the sucrose metabolism gene sequences in the *Solanum lycopersicum* and *Vitis vinifera* used in this study.
